# A digital twin for Escherichia coli K1 neonatal meningitis

**DOI:** 10.1099/jmm.0.002143

**Published:** 2026-03-16

**Authors:** Ruqaiyyah Siddiqui, Naveed Ahmed Khan

**Affiliations:** 1 Microbiota Research Center, Istinye University, Istanbul, 34010, Turkey; 2 Institute of Biological Chemistry, Biophysics and Bioengineering, Heriot-Watt University Edinburgh, Edinburgh EH14 4AS, UK; 3 School of Science, College of Science and Engineering, University of Derby, Derby, DE22 1GB, UK

**Keywords:** bacteria, blood–brain barrier (BBB), brain, central nervous system (CNS), digital twin, meningitis

## Abstract

*Escherichia coli* K1 is a major Gram-negative pathogen responsible for neonatal meningitis. Despite significant progress in antimicrobial therapy and neonatal intensive care, clinical outcomes remain problematic due to delayed diagnosis, rapid disease progression and a lack of precision tools for personalized management. Here, we propose a technical and translational digital twin framework for *E. coli* K1 infection in neonates that integrates clinical, microbiological, physiological and molecular data within a continuously adaptive computational model. This twin would simulate bloodstream invasion, blood-brain barrier traversal and central nervous system inflammation in real time, enabling dynamic prediction of disease and optimization of antibiotic regimens. The framework is intended as a technical resource for clinicians and modellers working in neonatal infectious disease. A digital twin may advance neonatal infectious disease management, i.e. transforming empirical treatment into evidence-based, patient-specific precision care while providing new mechanistic insights into host-pathogen interactions.

## Introduction

Neonatal meningitis remains one of the most challenging infections in paediatric medicine, with *Escherichia coli* K1 accounting for nearly half of all Gram-negative cases worldwide[1,3]. Neonatal bacterial meningitis affects ~0.2-0.3 per 1,000 live births globally, with mortality between 10 and 25% and up to half of survivors experiencing long-term neurological complications [4,6]. The pathogenesis involves maternal colonization, perinatal transmission, bacteraemia and ultimately traversal of the blood-brain barrier (BBB) leading to the central nervous system (CNS) infection [7,8]. Despite advances in antimicrobial chemotherapy, morbidity and mortality remains significant, with survivors often suffering from seizures, hydrocephalus, cerebral palsy or cognitive impairment [4,6,9]. Current management strategies rely on antibiotic regimens; however, efficacy is variable, and therapeutic drug monitoring is rarely performed [10]. Conventional infection models, although informative at the population level, fail to capture infection complexities in an individual neonate [11,12]. Infection models include *in vivo* neonatal infection models such as rodent meningitis models, *in vitro* systems such as brain microvascular endothelial cell monolayers and emerging three-dimensional engineered BBB models [13,14], which, although informative at the population level, still fail to capture the patient-specific complexities of infection in an individual neonate. This translational gap highlights the need for a technical framework that can integrate clinical, microbiological and pharmacological data into patient-specific models of *E. coli* K1 disease progression to represent the dynamic interplay between pathogen behaviour, host response and treatment kinetics at the level of a single patient.

The emerging concept of digital twins, i.e. computational replicas of real systems that evolve through continuous data assimilation, offers a novel route to individualized infection modelling. Digital twins have been applied to cardiology and oncology [15,18]. More recently, digital twins have been introduced in infectious disease contexts, including Coronavirus Disease 2019 (COVID-19) and antimicrobial resistance management [19,20]. Digital twin frameworks have also been proposed in infectious disease contexts such as amoebic neuroinvasion, alongside emerging applications in perinatal and neonatal care, including preterm birth prediction and systems-level neonatal modelling [21,23, 24]. Digital twins can integrate physiological, genomic and phenotypic data to transform personalized care, disease modelling and health-system operations [25]. These studies demonstrate that integrating clinical, immunological and pharmacological data within continuously adaptive computational models can enable real-time prediction of infection and therapeutic outcomes. *E. coli* K1 meningitis demands such an approach. Its rapid progression, high interindividual variability and multifactorial pathophysiology involving bacterial invasion, BBB permeability and neonatal immune immaturity make it uniquely suited to a digital twin. By integrating microbiological, physiological and pharmacokinetic data, a digital twin could provide real-time insight into disease evolution and guide personalized antibiotic strategies, transforming empirical care into precision neonatal medicine.

Pathogenesis begins with colonization of the neonatal gut by *E. coli* K1, followed by haematogenous spread and invasion of the CNS at the BBB [26] using a variety of virulence factors, including outer membrane protein A (OmpA), IbeA, IbeB, cytotoxic necrotizing factor 1 (CNF1) and FimH adhesins [27]. Bacterial invasion of the endothelial cell monolayer that constitutes the BBB is a key step in *E. coli* K1 penetration of the CNS [28,29]. After initial endothelial attachment, K1 triggers cytoskeletal rearrangements in brain microvascular endothelial cells through activation of MAPK, PI3K/Akt and NF-κB pathways. Simultaneous secretion of pro-inflammatory cytokines (IL-6, IL-8 and TNF-α) increases permeability, facilitating bacterial traversal into the CNS. Within the CNS, the organism multiplies rapidly, provoking inflammation that drives neuronal injury, excitotoxicity and apoptosis [11,30,32]. Host factors such as reduced microglial and complement systems, neutrophil activity and limited opsonization capacity further exacerbate vulnerability, which is highly variable among patients [1]. This variability underscores the need for integrative, data-rich modelling capable of representing molecular, cellular and systemic dimensions of disease that can be addressed using a digital twin.

## Framework and technical overview

**Fig. 1.**
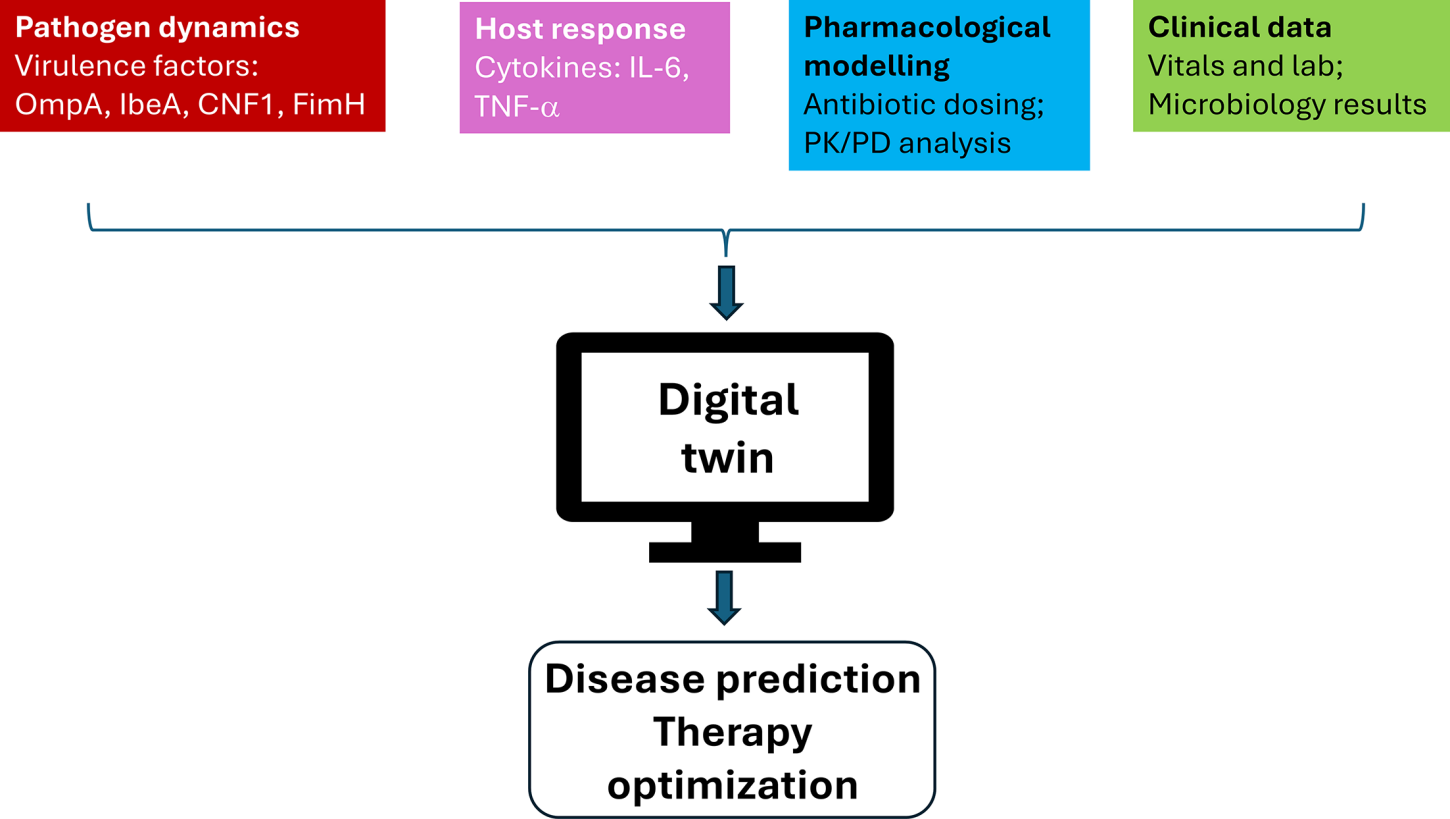
Conceptual approach for *E. coli* K1 neonatal meningitis digital twin. The digital twin integrates pathogen, host, pharmacological and clinical data within a continuously adaptive model. Pathogen inputs capture bacterial burden and virulence activity, host inputs reflect inflammatory and endothelial responses, and pharmacological inputs represent antibiotic dosing and exposure. Clinical data include vital signs, laboratory results and microbiology findings. These data are assimilated in real time to generate probabilistic predictions of disease progression and support personalized antibiotic management.

**Fig. 2.**
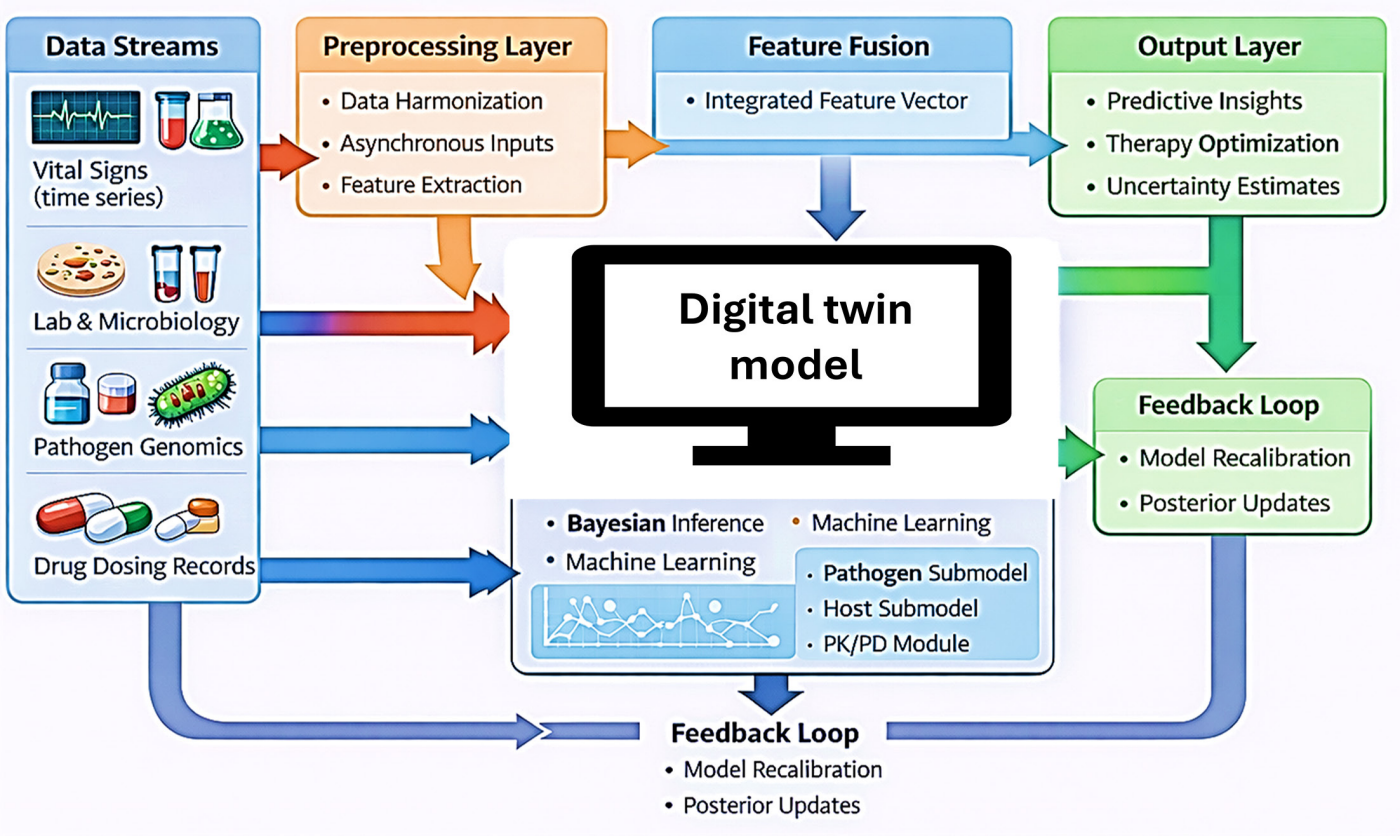
Workflow of *E. coli* K1 digital twin. Clinical, microbiological, physiological and pharmacological data streams are aligned through preprocessing and integrated across pathogen, host, pharmacological and clinical modules. A data-assimilation layer updates the model as new information becomes available, generating real-time predictions of disease progression and treatment response.

The proposed *E. coli* K1 neonatal meningitis digital twin is organized as a modular computational framework that integrates pathogen behaviour, host response, treatment effects and clinical physiology at the level of an individual neonate. Its architecture includes four core components, and an overview is illustrated in Fig. 1. A proposed workflow schematic outlining the data integration and model architecture is presented in Fig. 2.

### Pathogen dynamics

This module models bacterial proliferation, expression of key virulence factors (e.g. OmpA, IbeA/B, CNF1 and FimH), bloodstream behaviour and mechanisms of BBB traversal based on microbiological and molecular datasets.

### Host response

This layer represents endothelial activation, cytokine signalling, BBB permeability changes and features of neonatal immune immaturity. It incorporates measurable clinical markers alongside latent inflammatory states.

### Pharmacological modelling

Antibiotic dosing, pharmacokinetics/pharmacodynamics and compartmental drug penetration profiles are integrated to simulate antimicrobial activity across plasma, BBB and cerebrospinal fluid compartments.

### Clinical physiology

Vital signs, laboratory findings, microbiology results and cerebrospinal fluid indices are assimilated as time-varying clinical inputs to maintain alignment with the evolving bedside picture.

These components are linked through a real-time data assimilation layer. Bayesian inference and machine-learning approaches update model parameters and latent variables whenever new clinical or laboratory information becomes available, enabling probabilistic prediction of disease progression and response to therapy. Validation follows a phased approach: retrospective calibration using historical neonatal datasets, prospective silent-mode operation and eventual pilot deployment. Interoperability with electronic health records and microbiology systems is supported through established clinical data standards. Together, this framework provides a concise technical foundation for modelling *E. coli* K1 neonatal meningitis, enabling personalized simulation and translational investigation across biological scales.

 Patient-specific digital twins function as a living computational model that mirrors the infection trajectory of each neonate with suspected or confirmed *E. coli* K1 sepsis or meningitis [19,33,35]. Its purpose is twofold: first, to provide predictive insight into disease progression; and second, to support real-time optimization of therapy based on patient-specific dynamics. At its foundation, the twin continuously assimilates multimodal data streams from bedside monitors, laboratory systems and microbiology platforms [19,20, 36]. The model also captures antibiotic administration records to allow direct linkage to pharmacological submodels [37,38]. Overlaying this mechanistic core, Bayesian inference and machine learning modules provide adaptive calibration [35,37, 39]. Each new observation, whether a change in vital signs, laboratory values or culture results, updates the posterior probability distribution of key latent states such as bacterial burden or BBB permeability. This maintains synchrony between model predictions and bedside reality. Through this continuous updating, the twin transitions from a generic simulation to a highly individualized representation of the patient’s disease process. The system quantifies uncertainty, providing credibility intervals rather than deterministic recommendations. Ultimately, the twin functions as a decision-support companion, augmenting clinical judgement with data-driven foresight [20,36].

Developing a clinically robust digital twin necessitates iterative validation. In the retrospective phase, the model would be trained and tested on historical datasets from neonatal intensive care units (NICUs) containing confirmed cases of *E. coli* K1 and other neonatal sepsis pathogens [19,20, 33, 35, 37]. This phase ensures that the model can reproduce observed infection trajectories and antibiotic responses. In the prospective phase, the twin would run in parallel with routine care, silently taking live data without influencing decisions. Discrepancies between predicted and actual outcomes would inform recalibration. The clinical deployment phase would involve controlled pilots within NICUs. Key endpoints would include time to effective therapy, duration of antimicrobial exposure, rates of treatment failure and clinical outcomes such as survival and neurodevelopmental status. Success could be defined by accurate replication of infection trajectories, earlier identification of clinical deterioration and improvements in time to effective therapy. To minimize automation bias, the twin could present uncertainty intervals and confidence ranges alongside all model outputs to support clear clinical interpretation. A staged evaluation pathway, progressing from retrospective validation to silent-mode operation and then controlled pilot use, could provide a safe and transparent route towards clinical integration. Beyond immediate therapeutic benefit, the twin’s accumulated data could serve as a high-resolution research resource, providing mechanistic insights into neonatal immune dynamics and antibiotic pharmacology. Integration with hospital systems would follow established interoperability standards ensuring exchange of data between the electronic health record, microbiology information systems and bedside monitoring equipment.

*E. coli* K1 digital twin can function as a bridge between fundamental microbiology and clinical translational science. Its most immediate application lies in precision therapeutics tailoring antimicrobial regimens to the pharmacological, immunological and physiological characteristics of individual neonates. However, its broader potential extends to drug development, clinical trial simulation and global health. In the research domain, the model could serve as a virtual laboratory, enabling *in silico* experiments on pathogen-host interactions, BBB modulation and novel adjunctive therapies. For instance, anti-adhesion molecules targeting OmpA or IbeA could be computationally tested for their impact on bacterial invasion rates before clinical evaluation. Similarly, anti-inflammatory or neuroprotective agents could be screened through the twin’s mechanistic modules, narrowing the translational gap between bench and bedside. From a public health perspective, aggregated twins across populations could reveal patterns of antimicrobial resistance, regional variations in pathogen virulence and healthcare inequalities. Such federated learning frameworks would allow model improvement without compromising patient privacy with each institution maintaining local data while contributing to shared global insights. At the health system level, digital twins have already been applied to infectious disease surveillance and outbreak management through spatial mapping of patient and clinician movements in hospital environments, as demonstrated by the 3D-DOSS system [40]. These spatial digital twins highlight how real-time integration of clinical, laboratory and environmental data can strengthen infection control and complement patient-level twins by linking bedside events to broader hospital epidemiology.

It is important to note that deploying digital twins in neonatal settings raises ethical and regulatory considerations; hence, data protection must adhere to the highest standards. Regulatory frameworks such as the UK’s Medicines and Healthcare products Regulatory Agency AI assurance guidelines and the EU’s proposed Artificial Intelligence Act provide essential guardrails for clinical-grade algorithms [33,41, 42].

The implications of developing a digital twin for *E. coli* K1 meningitis extend beyond neonatal care. The framework could be generalized to other neurotropic pathogens such as *Listeria monocytogenes*, Group B *Streptococcus* or parasitic and viral infections. Such models could enhance training for clinicians and scientists, offering interactive platforms to visualize complex host-pathogen dynamics. Cloud-based twins, trained on diverse global datasets, could provide decision support to clinicians in under-resourced settings, guided by locally relevant antibiograms and epidemiology. Collaboration between academic centres, health services and industry will be essential to realize these expectations.

In conclusion, the proposed *E. coli* K1 digital twin represents a next-generation approach to neonatal infectious disease: dynamic, integrative and inherently translational. By converging clinical observation, microbial genomics, immune profiling and pharmacological modelling, it creates a unified platform for understanding and managing neonatal meningitis with unprecedented granularity. Beyond its immediate clinical utility, it lays the groundwork for a new era of computational paediatrics, where mechanistic insight and data-driven prediction can support precision care from the earliest stages of life. The path forward will require multidisciplinary collaboration, transparent governance and a commitment to clinical and ethical rigour. Yet the potential rewards, including safer, faster and more effective treatment for one of the most devastating neonatal infections, justify the ambition. In connecting molecules to minds and data to decisions, the *E. coli* K1 digital twin exemplifies how computational medicine can reshape our understanding of infection and recovery in the newborn brain; however, intensive future research is needed to realize these expectations.

## Abbreviations

BBB: blood–brain barrier
CNF1: cytotoxic necrotizing factor 1
CNS: central nervous system
COVID-19: coronavirus disease 2019
NICUs: neonatal intensive care units
OmpA: outer membrane protein A
